# Exploring the Regulation of *Tmem182* Gene Expression in the Context of Retinoid X Receptor Signaling

**DOI:** 10.3390/jdb13040034

**Published:** 2025-09-24

**Authors:** Saadia Khilji, Munerah Hamed, Jihong Chen, Qiao Li

**Affiliations:** 1 Department of Cellular and Molecular Medicine, Faculty of Medicine, University of Ottawa, Ottawa, ON K1H 8L1, Canada; 2 Department of Pathology and Laboratory Medicine, Faculty of Medicine, University of Ottawa, Ottawa, ON K1H 8L1, Canada

**Keywords:** nuclear receptor signaling, Tmem182, myogenic regulation

## Abstract

We have previously established that bexarotene, a clinically approved agonist of retinoid X receptor (RXR), promotes the differentiation and fusion of skeletal myoblasts. We have also analyzed the genomic programs underlying rexinoid-enhanced myogenic differentiation to identify novel regulatory pathways. As such, we observed a significant upregulation of a transcript encoding a predicted transmembrane protein, Tmem182, during C2C12 myoblast differentiation. Despite the documentation of *Tmem182* expression in skeletal muscles, its regulation had yet to be explored. Here, we show that *Tmem182* gene expression is markedly augmented in early myoblast differentiation and further enhanced by RXR signaling. In addition, *Tmem182* expression is specific to muscle tissues and related to muscle master regulator MyoD. We found that MyoD and histone acetyltransferase p300 are bound to the *Tmem182* promoter, and *Tmem182* expression is p300-dependent. Thus, our data display a putative epigenetic signature associated with p300 and histone acetylation in rexinoid-responsive locus activation and transcription of myogenic targets.

## 1. Introduction

The proper formation of skeletal muscle requires sequential expression of muscle regulatory factors (MRFs), including Myf5, MyoD, myogenin and MRF4 [1,2,3,4]. MRFs are a group of basic helix–loop–helix (bHLH) transcription factors that bind a conserved DNA motif known as an E-box, present in the regulatory loci of many skeletal muscle-specific genes [5]. Myf5 and MyoD regulate proliferation and early differentiation of myoblasts, diminishing in expression soon after differentiation has begun [6]. Myogenin is critical for the regulation of late differentiation and fusion, and MRF4 is expressed several days after fusion [7]. Studies have also delineated additional functions for MyoD in terminal differentiation and of MRF4 in the commitment of muscle progenitor cells, suggesting that the proper development of skeletal muscle requires the coordinated interaction of MRFs within complex transcriptional regulatory networks [8,9,10].

Adult skeletal muscles have the ability to regenerate upon stress or injury due to the presence of quiescent adult muscle stem cells known as muscle stem cells (MuSCs), which are localized between the sarcolemma and basal lamina [11]. The paired-box transcription factor Pax7 is critical for the specification of these cells as its inactivation in mice results in the complete absence of muscle satellite cells [12]. Pax7 expression is found in proliferating myoblasts but is rapidly downregulated upon differentiation. MuSCs become activated in response to muscle stress or injury and begin to express MyoD and either maintain MyoD expression while downregulating Pax7 to differentiate and fuse into existing fibers or lose MyoD expression but maintain Pax7 expression to repopulate the MuSC niche [13]. This highly efficient process in normal skeletal muscle is often compromised in cases of skeletal muscle dystrophy or diseases.

Myogenic gene expression relies on the recruitment of histone acetyltransferases (HATs), such as p300, which functions as a transcriptional coactivator and is part of multi-protein complexes that are recruited to the regulatory regions of target genes [14]. Knockout studies in mice and embryonic stem cells have delineated that the HAT activity of p300, and not its functional homolog CBP, is crucial for skeletal myogenesis and for the expression of muscle-specific genes, such as Myf5 and MyoD [15,16,17,18]. Additionally, as a global coactivator of numerous cellular processes, including differentiation [19,20,21], p300 is a key chromatin signature of enhancers where H3K18 and H3K27 are its main acetylation targets [22,23,24,25]. Although, histone acetylation decreases globally during the course of myogenic differentiation, we have previously characterized an increase in loci- and residue-specific histone acetylation at p300-associated enhancers in early myoblast differentiation, particularly when it is recruited by MyoD [26,27]. Taken together, these data indicate that p300 is an important coactivator for myogenic expression and provide a valuable signature to identify novel regulatory elements for studies of transcriptional control.

Chromatin modifications have recently emerged as a key avenue to identify networks of regulatory loci that drive gene expression in cellular differentiation. While histone acetylation plays an important role in the activation of gene expression [28], histone methylation associates with both gene activation and repression, dependent upon the specific lysine residue and the state of methylation [29,30]. For example, actively transcribed genes are characterized by high levels of H3K4me3 and H3K36me3, whereas H3K27me3 is associated with transcriptional repression [31,32]. Furthermore, H3K9ac, H3K18ac, and H3K27ac are found in regions surrounding transcription start sites (TSS) in combination with H4K8ac at promoter regions [33,34]. Thus, genomic localization of chromatin modifications is a useful tool to identify regulatory regions and provide indications for activation of distinct gene programs.

The nuclear receptor (NR) superfamily of transcription factors broadly influence gene expression in response to steroids, lipids, and other small molecules or synthetic ligands [35]. Retinoid X receptor (RXR) belongs to the Type II NR family and has the ability to form heterodimers with one third of the other NRs, allowing it to mediate a large array of signaling pathways. Three different isoforms of RXR have been identified (α, β, and γ); however, RXRα is the predominant subtype expressed in adult skeletal muscle [36]. RXRα null mice die in utero due to myocardial malformations highlighting its critical role in development. As type II NRs, RXR dimers constitutively bind a consensus sequence in the promoters or enhancers of the genes they regulate but require the presence of specific ligands to initiate ligand-induced transcription [37]. We have previously reported that bexarotene, a selective RXR agonist, promotes the specification and differentiation of skeletal myoblasts through the function of RXRα [38,39]. Interestingly, we also found an association of myogenin with rexinoid-responsive gene expression coupled with residue-specific histone acetylation at enhancers co-occupied by p300 and myogenin [40].

Since RXR signaling distinctly promotes myogenic gene expression, we utilized condition-specific gene expression profiling in rexinoid-enhanced myoblast differentiation to characterize gene activation patterns induced during differentiation [41]. This led us to the identification of *Tmem182* as a novel myogenic target that is highly upregulated during myoblast differentiation. In the context of myogenesis, *Tmem182* was first described by Kuninger et al. in a study of novel genes induced during growth factor-mediated muscle cell survival and differentiation [42]. Follow-up studies have described a gradual upregulation of *Tmem182* in skeletal muscle from the embryonic stage to adulthood [43], with a striking ~770-fold increase in *Tmem182* transcript levels during in vitro differentiation of C2C12 myoblasts [44]. Recent work has established Tmem182 as a negative regulator of myoblast differentiation, fusion, and skeletal muscle regeneration, acting through its direct interaction with integrin beta 1 (ITGB1) and downstream signaling pathways, including the FAK-ERK and FAK-Akt axes [43]. Similarly, in cardiomyocytes, Tmem182 was shown to inhibit differentiation by maintaining the active state of Wnt/β-catenin signaling via integrin-linked kinase (ILK) [45]. Collectively, these findings suggest that Tmem182 leverages integrin-mediated pathways to suppress muscle differentiation. However, the precise mechanisms by which RXR signaling intersects with Tmem182 to influence myogenesis remain unclear. In this study, we aim to elucidate the regulation of *Tmem182* expression in RXR-enhanced myoblast differentiation, shedding light on its broader implications for skeletal muscle health and disease.

## 2. Methods

### 2.1. Cell Culture and Reagents

C2C12 myoblasts (CRL-1772) were obtained from the American Type Culture Collection (Manassas, VA, USA). Cells were maintained in DMEM supplemented with 10% FBS (GM) at 37 °C with 5% CO_2_. To induce differentiation, medium of 70–80% confluent cell cultures was changed to DMEM supplemented with 2% horse serum (DM). Bexarotene was purchased from LC Laboratories and UVI3003 from Tocris.

### 2.2. Immunofluorescence Microscopy

C2C12 cells were fixed with cold methanol, rehydrated in phosphate-buffered saline (PBS), and incubated with myosin heavy chain antibody (hybridoma MF20) overnight. The next day, cells were washed with PBS and incubated with Alexa Fluor 594 secondary antibody (Invitrogen, Carlsbad, CA, USA). The cells were also incubated with 0.1 μg/mL Hoechst to stain cellular DNA. Zeiss AxioObserver D1 Microscope and AxioVision Rel 4.6 software were used to capture images via a 10× objective. Five random images were analyzed for each coverslip. ImageJ software was used for cell counting. Student’s *t*-tests were used for the statistical analyses.

### 2.3. Western Analysis

Cells were washed with PBS, harvested, and centrifuged. The resulting pellet was resuspended and shaken at 4 °C for 30 min in whole-cell extraction (WCE) buffer containing 50 mM Tris-HCl (pH 7.6), 400 mM NaCl, 10% glycerol, 1 mM dithiothreitol, 1 mM phenylmethylsulfonyl fluoride, and 1% Nonidet P-40 (NP-40). The lysates were centrifuged at 14,000× *g* at 4 °C for 10 min. Protein concentration was determined by a Bradford assay (Bio-Rad, Hercules, CA, USA) and quantified with Multiscan Spectrum photospectrometer (Thermo Fisher Scientific, Waltham, MA, USA). Equal amounts of protein were resolved on SDS-polyacrylamide gel and transferred onto an Immuno-Blot PVDF membrane (Bio-Rad). Bio-Rad ChemiDoc MP System was used to capture chemiluminescent images and ImageJ 1.53a software was used for densitometry quantification. All experiments were performed at least three times. The antibodies against Tmem182 and cyclophilin B were from Abcam (ab177360 and ab16045, respectively). Antibodies against myogenin (F5D) and β-tubulin (E7) were from Developmental Studies Hybridoma Bank.

### 2.4. Reverse Transcription PCR Analysis

Total RNA was isolated from C2C12 myoblasts using the GeneJET RNA Purification Kit (Thermo Fisher Scientific, Waltham, MA, USA) and equal amounts of RNA were reverse transcribed using a High-Capacity cDNA Reverse Transcription Kit (Applied Biosystems, Foster City, CA, USA). Real-time PCR was conducted in triplicates using a SYBR^®^ Green PCR Master Mix and HotStarTaq DNA polymerase (Qiagen, Carlsbad, CA, USA) on a CFX96 Touch Real-Time PCR Detection System (Bio-Rad, Hercules, CA, USA). Results were analyzed by the threshold cycle (Ct) comparative method using TATA-binding protein (Tbp) or Ribosomal protein S26 (Rps26) as an internal control. The experiments were repeated at least three times. Data were presented as the mean ± S.E.M, where *p* values were determined using Student’s *t*-test. The following primers were used: Tmem182 primer forward, AGTTCTGGTACACCAATCAGCC, and reverse, ATTGCGGAGTCGTAGGAGGT; myogenin primer forward, ATCCAGTACATTGAGCGCCTAC, and reverse, AGCAAATGATCTCCTGGGTTGG; myomaker primer [46] forward, ATCGCTACCAAGAGGCGTT, and reverse, CACAGCACAGACAAACCAGG.

### 2.5. Quantitative ChIP Analysis

Cells were crosslinked and sonicated followed by chromatin immunoprecipitation as previously described [18]. Following ChIP, purified DNA was amplified in triplicate real-time PCR reactions with locus-specific primers. A standard curve was created for each set of primers with input DNA, followed by quantification of the abundance of immunoprecipitated target DNA in relation to input chromatin DNA. Each qChIP was repeated at least three times. Antibodies against p300 (sc-584x), MyoD (sc-32758x), and Myogenin (sc-12732) were obtained from Santa Cruz Biotechnology. Primer pairs used for qPCR amplification were: Tmem182 promoter forward, GGGAGCAAGCAACTCCTCAA and reverse, GAGCTGTGGTGGCTTCTCAT.

## 3. Results

### 3.1. Tmem182 Is a Rexinoid-Responsive Target and Expressed in Skeletal Muscle

**Figure 1:**
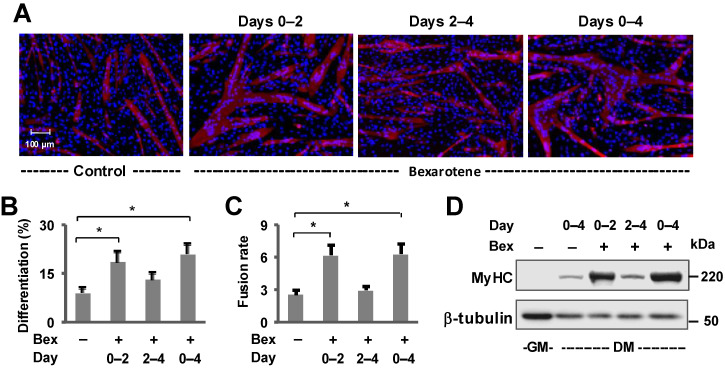
Bexarotene enhances differentiation and fusion of C2C12 myoblasts. (**A**) C2C12 myoblasts were differentiated for 4 days in the absence or presence of bexarotene (Control or Bex, 50 nM) for the time periods specified and stained for myosin heavy chain (red) and nuclei (blue). Representative microscopy images are shown. (**B**) Differentiation was defined as the percentage of myogenic nuclei relative to the total number of cell nuclei. (**C**) Fusion rate was defined as the number of nuclei per MyHC positive myotube. Error bars are the standard deviations of three independent experiments (*, *p* < 0.05). (**D**) MyHC protein levels were determined by Western blotting with β-tubulin as a loading control.

We have previously established that RXR signaling promotes the differentiation and fusion of skeletal myoblasts [39,40,41]. In this study, we used an interval approach to examine the window of rexinoid signaling in enhancing the myogenic program (Figure 1). As such, C2C12 myoblasts were differentiated for four days in the presence of bexarotene for different intervals: during the first two days of differentiation, the last two days or continuously throughout the four-day period. Intriguingly, only the addition of bexarotene during the first two days or throughout the four days of differentiation significantly enhanced the differentiation and fusion of myoblasts as determined by quantitative microscopy (Figure 1A–C). Two days after the initiation of myoblast differentiation, bexarotene treatment failed to significantly augment the differentiation or fusion of myoblasts compared to treatment during the first two days or throughout the 4 days of differentiation (Figure 1A–C). Additionally, the ability of bexarotene to enhance myogenic differentiation was also illustrated by a significant increase in muscle marker, MyHC compared with untreated myoblasts in the Western blot analysis (Figure 1D, Figure S1), suggesting that bexarotene treatment is most efficient during the early stage of differentiation as it may enhance myogenesis via an augmentation of early gene expression [41].

**Figure 2:**
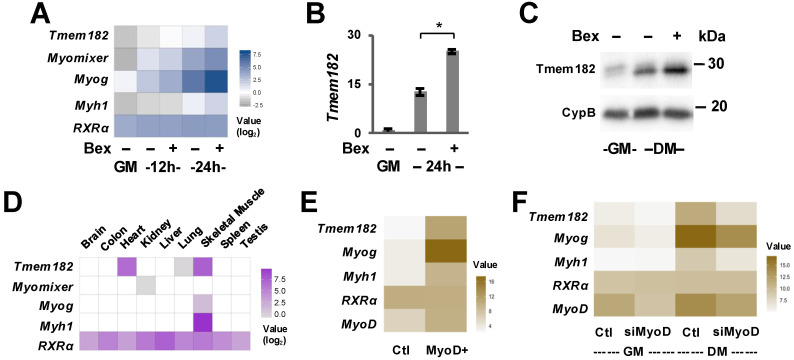
Tmem182 is expressed in skeletal muscle. (**A**) The heatmap displays expression profiles for the indicated genes as determined by RNA-seq analysis [GSE94560] of proliferating C2C12 myoblasts (GM) and those differentiated in the absence or presence of bexarotene (Bex, 50 nM) for 12 or 24 h. (**B**) The expression levels of *Tmem182* were determined by RT-qPCR in primary myoblasts differentiated in the absence or presence of bexarotene (30 nM) for 24 h. Expression values are presented as fold change relative to proliferating myoblasts (error bars: SEM; *n* = 3; *, *p* < 0.05). (**C**) Western analysis of Tmem182 and cyclophilin B (CypB) following 2 days of differentiation (DM) in C2C12 myoblasts. (**D**) The heatmap presents expression levels collected from strand-specific RNA-seq (FPKM) for nine mouse tissues in the CD1 strain with an expression level cut-off of 0.5 [GSE41637]. (**E**) Gene expression data from microarray analysis on C3H/10T1/2 fibroblasts in untreated control (Ctl) and 48 h following myogenic conversion and 24 h after inducing differentiation (MyoD+). (**F**) Gene expression values obtained from microarray analysis of proliferating C2C12 myoblasts (GM) and myoblasts differentiated for 24 h (DM) subjected to siRNA-mediated knockdown of MyoD or a non-silencing control [GSE66319].

Since bexarotene promotes early myogenic gene expression, we utilized selective ligand-induced activation of myogenic programs to delineate novel myogenic targets and molecular interactions underlying skeletal myogenesis [41]. We have analyzed differential gene expression in C2C12 myoblasts differentiated for 12 or 24 h in the absence or presence of bexarotene with proliferating myoblasts as control. From the list of genes with unknown functions in myogenesis, we focused upon Tmem182, a novel transmembrane protein that is upregulated nearly 5-fold during the first 24 h of differentiation (Figure 2A), similar to other muscle-specific membrane protein such as myomixer [47,48,49]. RT-qPCR and Western blotting analysis validated that the levels of *Tmem182* mRNA and protein were significantly augmented further following the addition of bexarotene (Figure 2B,C, Figure S1). Notably, bexarotene enhanced Tmem182 expression by approximately 1.85-fold at 12 h and 2.5-fold at 24 h compared to untreated cells at the same time points. Due to the lack of functional information regarding Tmem182, we also assessed its tissue-specificity across nine mouse tissues [50], which displayed the highest Tmem182 expression in muscle tissue including skeletal and cardiac muscle (Figure 2D). Thus, Tmem182 is presented as a rexinoid-responsive target with an expression profile similar to other important myogenic regulators such as myogenin.

### 3.2. The Regulation of Tmem182 Expression

As bexarotene enhances myoblast differentiation and fusion largely via a direct regulation of MyoD [41], we assessed the role of MyoD for *Tmem182* expression. As shown in Figure 2E, microarray data from overexpression of MyoD to induce myogenic conversion of C3H/10T1/2 fibroblasts [51] displayed a substantial upregulation of *Tmem182* expression, similar to other known gene targets of MyoD [47,48,49,52,53]. Interestingly, loss of MyoD in C2C12 myoblasts dramatically reduced *Tmem182* mRNA expression during the differentiation process but remained largely unaffected in proliferating conditions (Figure 2F). Collectively, these results indicate that *Tmem182* is upregulated during the differentiation process, and its levels may be regulated by differentiation-specific augmentation of MyoD (Figure 2).

**Figure 3:**
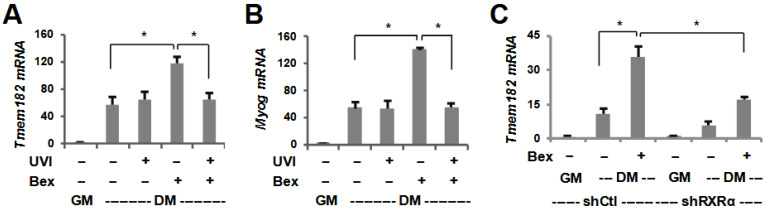
Role of RXR in bexarotene-enhanced myogenic expression. (**A**,**B**) C2C12 cells were differentiated in the absence or presence of bexarotene (Bex, 50 nM) and RXR antagonist UVI 3003 (1 μM) for 24 h (DM). The mRNA levels for *Tmem182* and *myogenin* were assessed by RT-qPCR and presented as the fold change relative to proliferating myoblasts (GM; error bars: SEM; *n* = 3; *, *p* < 0.05). (**C**) RXRα knockdown cells (shRXRα) were differentiated in the absence or presence of bexarotene for 24 h with a non-silencing shRNA used as control (shCtl). *Tmem182* expression levels were determined by RT-qPCR and presented as the fold change in relation to proliferating myoblasts.

**Figure 4:**
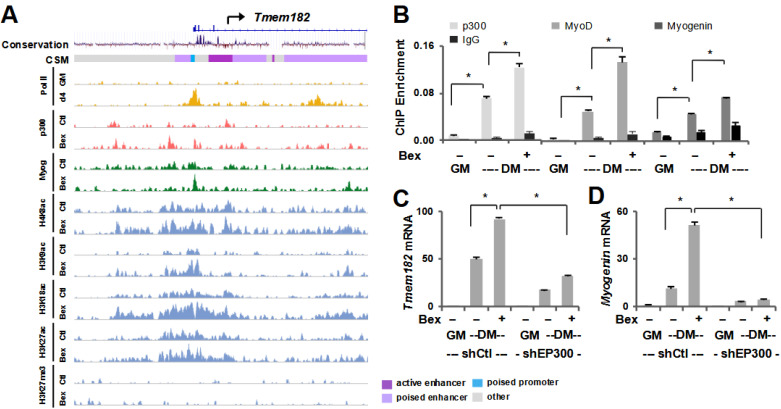
Regulation of Tmem182 expression. (**A**) C2C12 myoblasts were differentiated in the absence or presence of bexarotene (Ctl or Bex) for 24 h. Genome browser view for enrichment of p300 and various histone modifications [GSE94558, GSE139942] is shown at the *Tmem182* locus. RNA Polymerase II (Pol II) enrichment was obtained from proliferating C2C12 myoblasts (GM) and myoblasts differentiated for 4 days (d4) [GSE25308]. Blue bars show Refseq gene position, and the panel below presents mammalian conservation of the locus as quantified by the UCSC genome browser. (**B**) C2C12 cells were differentiated for 24 h (DM) in the presence or absence of bexarotene with proliferating myoblasts as controls. ChIP-qPCR analysis was performed at the *Tmem182* promoter using antibodies against p300, MyoD, and myogenin. Normal IgG antiserum and a random locus (Ctl) were used as negative controls. Quantification is presented as the percentage of enrichment in relation to input chromatin DNA (error bars: SEM; *n* = 3; *, *p* < 0.05). (**C**,**D**) *Tmem182* and *myogenin* mRNA levels were examined by RT-qPCR and presented as the fold change in relation to proliferating myoblasts following p300 knockdown (shEP300), with a non-silencing shRNA (shCtl) used as a control.

Given that *Tmem182* is a bexarotene-responsive gene target, we also assessed the requirement of RXR for *Tmem182* expression (Figure 3A,B). To this end, we utilized a known and potent RXR antagonist, UVI3003 [54], in that C2C12 myoblasts were differentiated with bexarotene in the absence or presence of UVI3003. Intriguingly, the RXR antagonist did not considerably affect *Tmem182* gene expression in untreated controls but significantly attenuated the enhancement effect of bexarotene on *Tmem182* and *myogenin* expression (Figure 3A,B). Furthermore, since bexarotene enhances myoblast differentiation via the activation of RXRα specifically, we utilized our established RXRα shRNA knock down stable myoblasts for the Tmem182 study [39]. As shown in Figure 4C, the induction of *Tmem182* by bexarotene was significantly attenuated in shRXRα stable myoblasts compared with the shRNA control myoblasts (Figure 3C). Interestingly, the loss of RXRα in the absence of bexarotene treatment also decreased Tmem182 expression compared to control myoblasts, suggesting a possible role for unliganded RXR in the expression of Tmem182, as it may disrupt RXR-mediated chromatin modifications at regulatory loci. Taken together, *Tmem182* is a target of RXR signaling in the enhancement of myogenic differentiation via RXRα as a transcription factor (Figure 3).

### 3.3. Tmem182 Expression Is p300-Dependent

Having identified *Tmem182* as a putative gene target of MyoD in early myoblast differentiation, we delved further into its regulation as a rexinoid target gene. As ChIP-seq is widely used to analyze the association of transcriptional co-regulators and histone modifications within the genome, we analyzed ChIP-seq data for the HAT p300, myogenin and various histone modifications representative of promoter and enhancer markers including H3K9ac, H3K18ac, H3K27ac, and H4K8ac in C2C12 myoblasts differentiated in the absence or presence of bexarotene [40,41]. Interestingly, the promoter region of *Tmem182*, signified by increased enrichment of RNA Polymerase II (Pol II) in myotubes, also displayed increased enrichment of p300 as well as myogenin following the addition of bexarotene in early differentiation (Figure 4A,B). Relatedly, the histone acetylation marks generally associated with gene expression were enriched around the TSS in differentiating myoblasts but were highly augmented following the addition of bexarotene, particularly H3K9ac and H3K18ac. Notably, the levels of H3K27me3, a polycomb repressive mark associated with repressed chromatin, were almost negligible in both conditions. Additionally, since mammalian conservation scoring displays a highly conserved region across vertebrate species including humans, the locus immediately upstream of *Tmem182*, characterized by an enrichment of gene activation related modifications, is likely an important putative regulatory region (Figure 4A).

As rexinoid-responsive gene expression is linked to residue-specific histone acetylation and an epigenetic signature related to histone acetylation [40], we also examined the role of p300 in the regulation of *Tmem182*. We employed p300 qChIP to quantify the enrichment of p300 at the promoter region of *Tmem182*. As seen in Figure 4B, p300 association to the *Tmem182* locus was significantly enriched following 24 h of differentiation and further increased markedly by bexarotene. This pattern paralleled the enrichment of MyoD and myogenin, suggesting that MRFs and p300 may act in concert to regulate Tmem182 expression during rexinoid-enhanced myogenic differentiation. (Figure 4B) We also used a previously established p300 shRNA knockdown myoblast system [55] to determine the requirement of p300 for *Tmem182* expression. As shown in Figure 4C, Tmem182 expression was considerably induced in shRNA control differentiating myoblasts and augmented further by bexarotene. However, shRNA knockdown of p300 attenuated *Tmem182* gene expression in differentiating myoblasts regardless of treatment, suggesting a critical role for p300 in *Tmem182* gene regulation and for its rexinoid-mediated augmentation, similar to the regulation of muscle-specific marker, *myogenin* (Figure 4D). Therefore, *Tmem182* expression is dependent upon the activities of MyoD and p300 in early myogenic differentiation.

## 4. Discussion

We have previously established that a selective RXR agonist, bexarotene, enhances the differentiation and fusion of myoblasts through a direct regulation of MyoD [39,41]. Here we show that bexarotene exerts its positive effects on myoblast differentiation through the first 48 h of differentiation specifically such that rexinoid signaling augments MyoD associated myogenic pathways during the early stages of differentiation (Figure 1). Importantly, we utilized the global profiling of transcriptional regulators underlying rexinoid-enhanced myogenic expression to identify novel myogenic targets, leading us to a transmembrane protein named Tmem182 (Figure 2). We demonstrate that *Tmem182* is a rexinoid-responsive and muscle-specific target that is upregulated during the differentiation process (Figure 3). Additionally, we delved into the regulation of *Tmem182*, which suggests that p300 and MyoD may cooperate for *Tmem182* expression within the context of rexinoid-responsive myogenic differentiation (Figure 4). Thus, we present Tmem182 as a novel target of p300 and MyoD in early myoblast differentiation and show that the model of bexarotene-enhanced myogenic differentiation is a powerful tool for identifying additional myogenic target genes and specific interactions that could be leveraged for developing therapeutics aimed at muscle regeneration and repair.

In this study, we have used C2C12 myoblasts to represent precursors of skeletal myocytes. The C2C12 myoblast cell line consists of a pure population of myoblasts which are predetermined to differentiate and fuse with each other into myotubes [56]. Following the induction of differentiation, myotubes start to appear by 24 h of differentiation and are evident by 48 h [57]. We have studied the role of RXR signaling in myoblast differentiation by using a treatment interval approach to compare myoblasts that are differentiated in the presence of bexarotene during the first 2 days of differentiation or between day 2 and day 4 (Figure 1). Our data clearly indicate that bexarotene enhances myoblast differentiation by targeting early myogenic transcription and is not as effective after the first 2 days of differentiation. Comprehensive gene expression analyses have presented a step-wise transcriptional blueprint of myogenic differentiation where thousands of genes are differentially expressed at the early stage of the differentiation, mostly within the first day of differentiation onset [41,58,59]. Thus, this time period encapsulates epigenetic changes reflecting the alteration of myogenic gene programs and the temporal waves of gene expression that are induced prior to the transcriptional activation of myogenin which initiates terminal differentiation [60]. Therefore, the addition of bexarotene is critical at the onset of differentiation to achieve effective enhancement of differentiation and fusion via the regulation of early myogenic genes.

Utilizing bexarotene enhanced transcriptome profiling, we have identified *Tmem182* as a target in early myoblast differentiation (Figure 2) and confirmed findings from previous studies that *Tmem182* is highly expressed in skeletal muscle [43]. Since *Tmem182* displays a differentiation-dependent upregulation in skeletal myogenesis as well as adipogenesis [44], its function may be related to cellular pathways shared by both lineages [44]. In vivo studies suggest that altered Tmem182 activity can influence muscle regeneration after injury and may contribute to the impaired repair seen in muscular dystrophies, where integrin–laminin interactions are already compromised [43]. Moreover, as *Tmem182* expression is enriched in adipose tissue and responds dynamically to nutritional and inflammatory cues, it may also contribute to systemic effects on body weight and energy balance [44]. These observations raise the possibility that Tmem182 functions at the intersection of muscle and metabolic homeostasis, and that its modulation could have therapeutic relevance not only for dystrophic muscle but also for conditions such as obesity and cachexia. Additionally, we analyzed published microarray data for C3H/10T1/2 fibroblasts induced to differentiate following MyoD overexpression, as well as a siRNA knockdown of MyoD in differentiating C2C12 myoblasts [51]. Our studies indicate that MyoD plays a regulatory role in *Tmem182* expression (Figure 2). Supporting this, previous research has shown that MyoD directly binds a conserved E-box in the *Tmem182* promoter and induces *Tmem182* transcription during myoblast differentiation [43]. Interestingly, the enrichment of RNA polymerase II at the *Tmem182* promoter following 4 days of differentiation (Figure 4) indicates a requirement for the protein up to the late stages of myogenesis, aligning with its functional capacity to modulate muscle differentiation and regeneration [43]. Although our study focused on skeletal myogenesis, Tmem182 has likewise been shown to inhibit differentiation in cardiomyocytes by sustaining Wnt/β-catenin activity through integrin-linked kinase, indicating a conserved role in striated muscle [43,45]. Given that RXRα is essential for cardiac development [61,62], and Tmem182 is rexinoid-responsive in skeletal muscle, it is plausible that RXR-dependent transcriptional programs intersect with Tmem182-mediated integrin signaling in both skeletal and cardiac contexts. However, further studies will be needed to outline its exact functional complexities and explore its potential as a therapeutic target in muscle-related diseases.

In addition to the functional importance of Tmem182, we also delineated the mechanisms by which its expression is regulated (Figure 3 and Figure 4). We examined the role of RXR for *Tmem182* expression as Tmem182 is a rexinoid-responsive target and bexarotene enhances myogenic differentiation through the activation of RXR. Interestingly, both antagonism of RXR and loss of RXR protein attenuated bexarotene-mediated enhancement of *Tmem182* expression, confirming that *Tmem182* is a target of RXR signaling in early myoblast differentiation. Nonetheless, there is no apparent RXR binding at the *Tmem182* locus [GSE94558]. Thus, *Tmem182* may be an indirect target of RXR, and bexarotene enhanced *Tmem182* expression is likely mediated via MyoD [41], which directly binds a conserved E-box in the *Tmem182* promoter to drive its transcription during myoblast differentiation (Figure 2 and Figure 4).

Since histone acetylation provides a useful baseline for gene expression, we also profiled the pattern of histone acetylation in C2C12 myoblasts differentiated in the absence or presence of bexarotene at the *Tmem182* promoter. Our results showed a distinct enrichment of histone acetylation particularly of H4K8, H3K9 and H3K18 following bexarotene treatment. This increase in histone acetylation was paralleled by enhanced recruitment of p300 as well as the myogenic regulators MyoD and myogenin (Figure 4). The combined enrichment of MRFs and p300 at the Tmem182 locus supports a cooperative mechanism for rexinoid-responsive transcription. Consistent with this, loss of p300 reduced Tmem182 expression and blunted its bexarotene-mediated augmentation. Since p300 is recruited by MyoD to myogenic loci where it acetylates histones and promotes the full transcriptional activity of MyoD on chromatin-associated templates [63], RXR-selective signaling may exploit this established transcriptional axis to enhance the expression of Tmem182 and other myogenic genes. Notably, the reduced expression of myogenin in p300-deficient cells suggests that the observed effects may not be solely due to impaired transcriptional activation but may also reflect a delay or disruption in the overall progression of the differentiation process. Together, these findings are in line with our previous observation that RXR signaling augments residue-specific histone acetylation at loci co-occupied by p300 and myogenic regulators such as MyoD and myogenin [40,41], with Tmem182 representing a specific example of this epigenetic signature. This cooperative regulation highlights how RXR signaling can shape the chromatin landscape at muscle-specific loci and may be relevant for contexts where myogenic regulators are implicated, including processes of skeletal and cardiac muscle development and repair.

In summary, our study introduces a novel myogenic target and explores the regulation of a previously uncharacterized locus which is used as a model to provide molecular insights into how p300 and histone acetylation is linked to bexarotene-responsive locus activation and transcription of myogenic targets. To our knowledge, this study provides the first insights into the rexinoid-responsive regulation of *Tmem182*, presenting evidence for its important role in myoblast differentiation. Future studies will be needed to investigate its roles in skeletal muscle development, regeneration, and dystrophy, which may provide a potential target for therapeutics design towards skeletal muscle health and disease.

## Data Availability

All data generated or analyzed during this study are included in this published article.

## Supplementary Materials

The following supporting information can be downloaded at https://www.mdpi.com/article/10.3390/jdb13040034/s1: Figure S1: Uncropped Western blot images corresponding to the experiments in the main text are shown at multiple exposure times. MyHC and β-tubulin blots correspond to the cropped panels in Figure 1D, and Tmem182 and cyclophilin B blots correspond to those in Figure 2C.
