# Supplementary material for: Exploring the Regulation of Tmem182 Gene Expression in the Context of Retinoid X Receptor Signaling
Source: J Dev Biol. 2025 Sep 24;13(4):34. doi: 10.3390/jdb13040034 (PMC12550946; doi:10.3390/jdb13040034)
Supplement: Supplementary file 1 [file jdb-13-00034-s001.zip › jdb-3814992-supplementary.pdf]

Figure S1: Uncropped Western blot images corresponding to the experiments in the main text are shown at multiple exposure times. MyHC and β-tubulin blots correspond to the cropped panels in Figure 1D, and Tmem182 and cyclophilin B blots correspond to those in Figure 2C.

MyHC Western blot

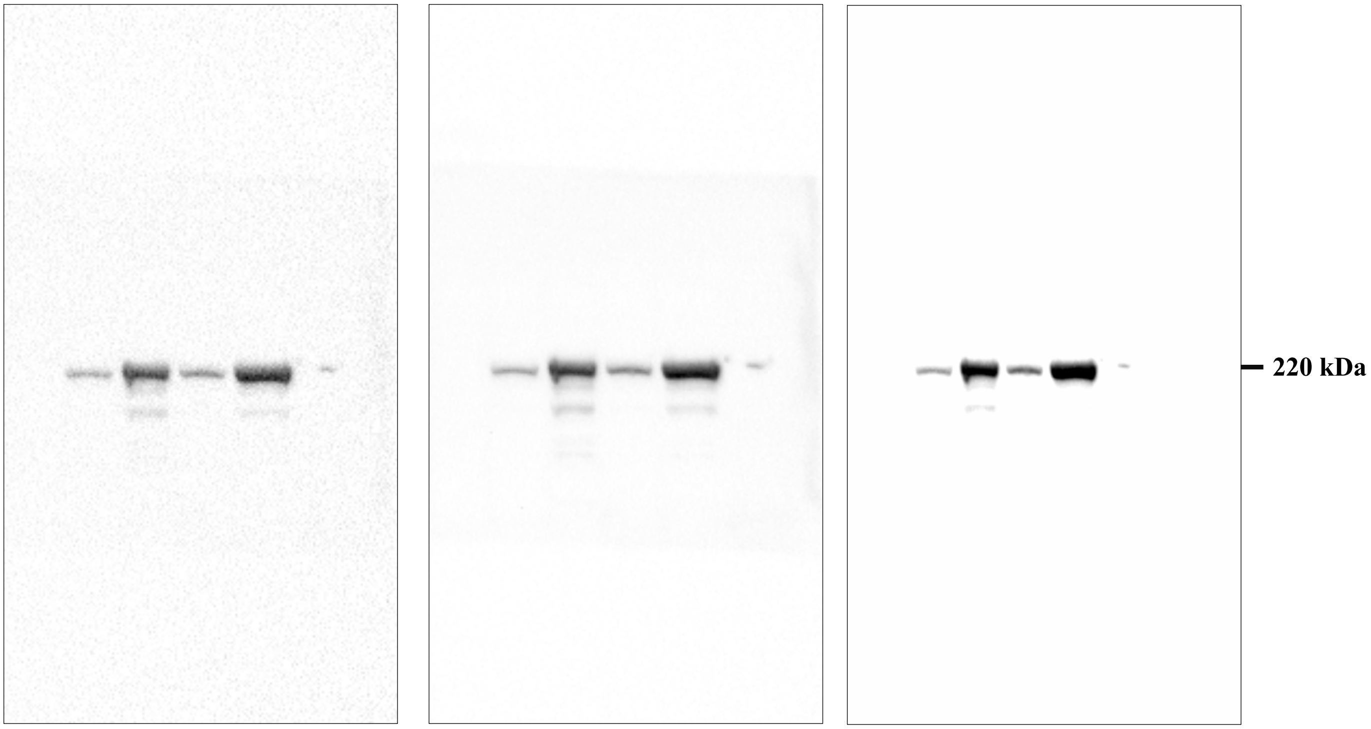

B-tubulin Western blot

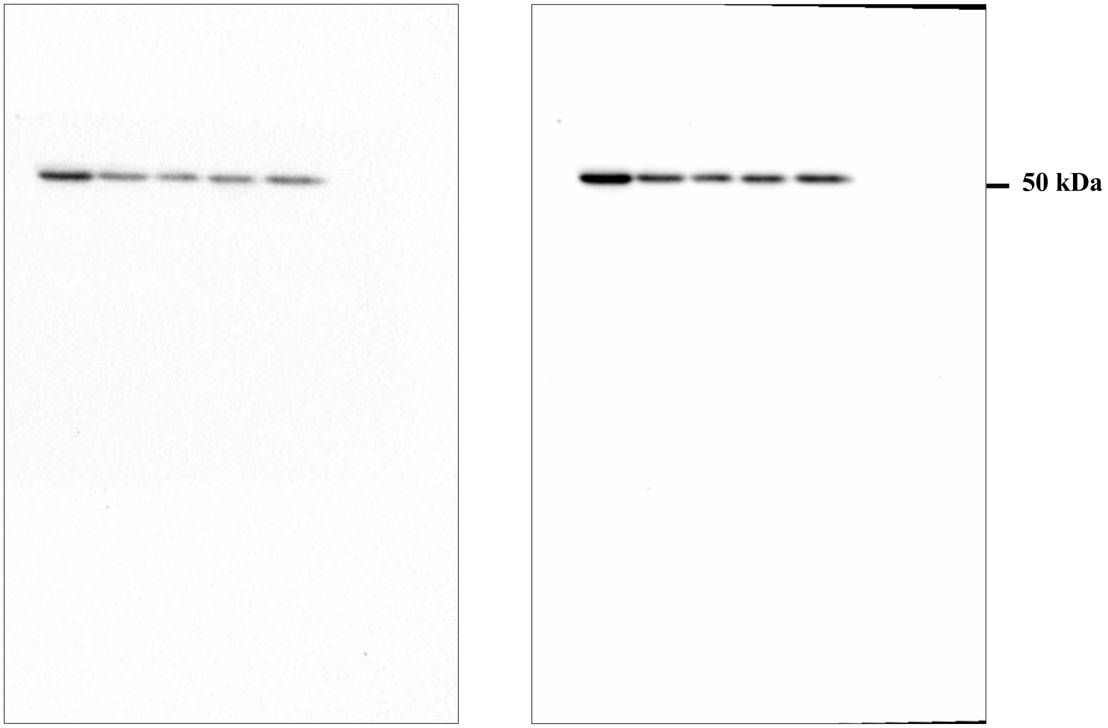

Tmem182 Western blot

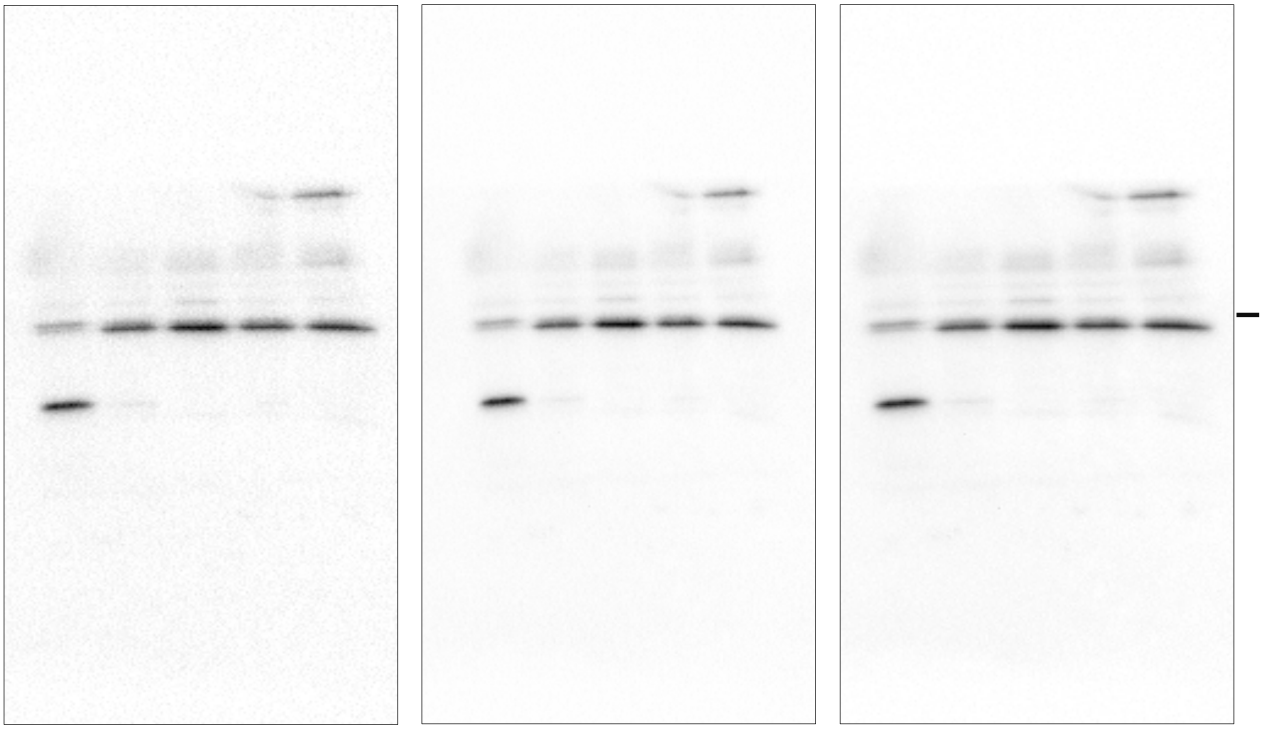

30 kDa

Cyclophilin Western blot

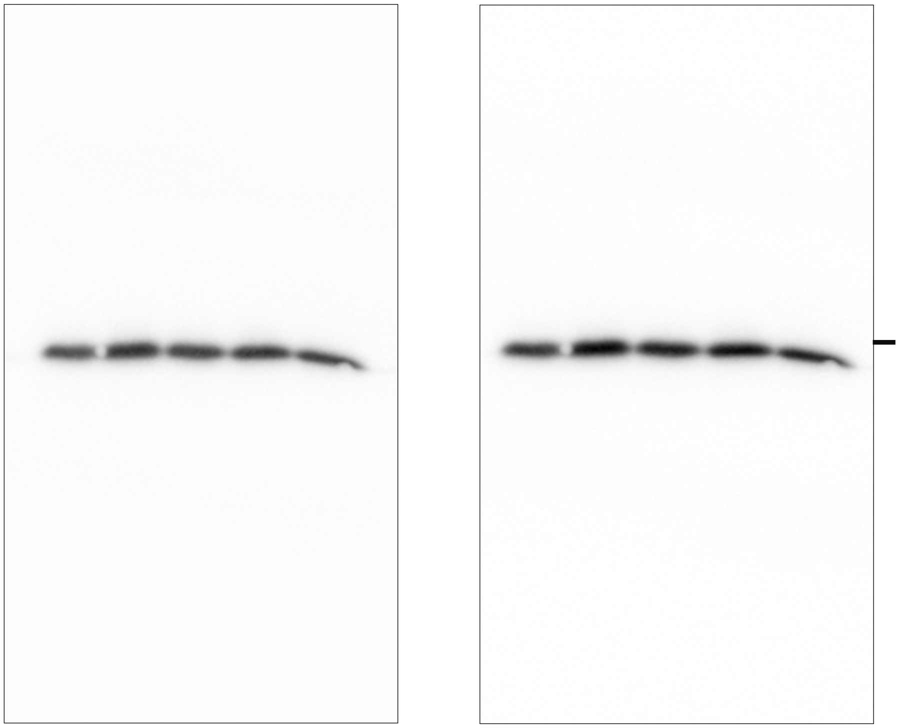

20 kDa
